# Progression of Postprandial Blood Plasma Phospholipids Following Acute Intake of Different Dairy Matrices: A Randomized Crossover Trial

**DOI:** 10.3390/metabo11070454

**Published:** 2021-07-14

**Authors:** Rebekka Thøgersen, Ida Emilie I. Lindahl, Bekzod Khakimov, Louise Kjølbæk, Klaus Juhl Jensen, Arne Astrup, Marianne Hammershøj, Anne Raben, Hanne Christine Bertram

**Affiliations:** 1 Department of Food Science, Aarhus University, Agro Food Park 48, DK-8200 Aarhus N, Denmark; idaemilielindahl@gmail.com (I.E.I.L.); marianne.hammershoj@food.au.dk (M.H.); hannec.bertram@food.au.dk (H.C.B.); 2 Department of Food Science, University of Copenhagen, DK-1958 Frederiksberg C, Denmark; bzo@food.ku.dk; 3 Department of Nutrition, Exercise and Sports, University of Copenhagen, DK-1958 Frederiksberg C, Denmark; louisekjoelbaek@nexs.ku.dk (L.K.); ara@novo.dk (A.A.); ara@nexs.ku.dk (A.R.); 4 Arla Foods Amba, DK-8200 Aarhus N, Denmark; kljes@arlafoods.com; 5 Steno Diabetes Center Copenhagen, DK-2820 Gentofte, Denmark

**Keywords:** cheese, casein, phospholipidomics, food structure, milk phospholipids

## Abstract

Studies have indicated that the dairy matrix can affect postprandial responses of dairy products, but little is known about the effect on postprandial plasma phospholipid levels. This study investigated postprandial plasma phospholipid levels following consumption of four different dairy products that are similar in micro and macro nutrients, but different in texture and structure: cheddar cheese (Cheese), homogenized cheddar cheese (Hom. Cheese), micellar casein isolate with cream (MCI Drink) or a gel made from the MCI Drink (MCI Gel). The study was an acute randomized, crossover trial in human volunteers with four test days. Blood samples were collected during an 8 h postprandial period and the content of 53 plasma phospholipids was analysed using liquid chromatography-mass spectrometry (LC-MS). No meal–time interactions were revealed; however, for nine of the 53 phospholipids, a meal effect was found. Thus, the results indicated a lower plasma level of specific lyso-phosphatidylethanolamines (LPEs) and lyso-phosphatidylcholines (LPCs) following consumption of the MCI Gel compared to the MCI Drink and Hom. Cheese, which might be attributed to an effect of viscosity. However, further studies are needed in order to reveal more details on the effect of the dairy matrix on postprandial phospholipids.

## 1. Introduction

Dairy products are important sources of high-quality proteins, lipids, vitamins, and minerals (particularly calcium and phosphorus), and they constitute a significant contribution to diet quality because of their nutrient richness [1]. However, dairy products also contain considerable amounts of saturated fatty acid (SFA), which is commonly perceived as potentially harmful. Thus, consumption of high amounts of SFA is associated with increased blood LDL-cholesterol levels and risk of cardiovascular disease (CVD) [2]. Thus, while a reduction in SFA intake has been the cornerstone of dietary recommendations to decrease CVD risk, the validity of these recommendations has recently been questioned, as clear contradictions are evident [3]. Accordingly, studies have shown that the impact of SFA consumption on plasma lipids depends on the specific dietary source of SFA [4,5,6], and evidence exists that SFA contained in cheese matrices does not increase blood lipids as compared to other dairy matrices [7]. Although the exact causes for such different effects of SFA on plasma lipids are not yet fully understood, it is anticipated that the effect of the food matrix and physical/textural structures (‘food matrix effect’), in which fat is incorporated and consumed, plays a pivotal role [3,7,8]. Apparently, intrinsic components present in the cheese matrix, such as protein, peptides, phospholipids derived from milk fat globule membrane (MFGM), calcium and phosphorous, are likely to influence the absorption of SFA in the human body [5,7].

Even though the exact underlying mechanisms behind this food matrix effect remain to be deciphered, some potential mechanisms have been proposed. Backed up by evidence revealing that calcium content impacts lipid absorption from dairy [9,10], it has been put forward that an underlying food matrix effect entails a reduction in fat bioaccessibility by calcium due to the formation of insoluble calcium soaps [11,12,13]. In addition, it has recently been hypothesized that milk phospholipids may bind cholesterol and thereby attenuate its absorption [14]. This hypothesis is supported by studies in animal models showing that milk phospholipids are reducing intestinal absorption of cholesterol [15], and thereby affecting plasma and hepatic lipid metabolism [16,17,18]. Recently, a 4-week intervention study with polar lipids derived from milk was shown to decrease plasma cholesterol, plasma triglycerides and Apolipoprotein B48 in overweight postmenopausal women [14]. Nevertheless, human studies focused on unravelling the role of phospholipids when consumed in the context of a whole dairy product are sparse. Therefore, the present study investigated the impact of a dairy matrix of four nutrient-matched meals of dairy products with different textural and structural properties on postprandial phospholipid absorption kinetics. Using a targeted liquid chromatography (LC) tandem mass spectrometry (MS) method previously developed for milk [19], quantification of approximately 50 glycerophospholipid and sphingolipid species was performed to investigate the presence of postprandial plasma phospholipids following consumption of the dairy products and to provide new knowledge about the effects of the dairy matrix on postprandial phospholipid status. The present study is part of the interdisciplinary DAIRYMAT project. The experimental design and primary outcome parameters are presented in Kjølbæk et al. [20] and a thorough characterization of the dairy products is provided in Schmidt et al. (2020) [21].

## 2. Results

LC-MS data are presented as arbitrary units; data correction was attained via normalization of the batch-corrected quantifier area of a given lipid according to the batch corrected quantifier of the corresponding internal standard.

Multivariate data analysis was used as an exploratory approach to investigate the phospholipid content in plasma samples during the postprandial period and a principal component analysis (PCA) model was constructed. The first two components explained 60% of the total variation. The PCA scores plot revealed an effect of time during the postprandial period (Supplementary Material Figure S1). The corresponding loadings plot revealed that plasma samples taken approximately two hours or more after consumption of the test meals were associated with higher content of PEs and PCs (Supplementary Material Figure S2).

Table 1 and Table 2 and Supplementary Material Tables S1–S4 show the resulting mean values in plasma samples for LPE, LPC, CER, SM, PE and PC, respectively. No meal–time interactions were revealed for any of the phospholipids. For the following phospholipids, a meal effect was found: LPE 18:2 (*q* = 0.001), LPE 18:1 (*q* < 0.001), LPE 18:0 (*q* = 0.026), LPE 20:3 (*q* = 0.016), LPE 20:2 (*q* < 0.001), LPE 20:1 (*q* = 0.001) (Table 1), LPC 16:3 (*q* = 0.026), LPC 18:1 (*q* = 0.038), LPC 18:0 (*q* = 0.045) (Table 2). The majority of the phospholipids showed no strong and systematic changes during the postprandial period and some remained almost constant during the 8-h period. Nevertheless, 31 out of the 53 investigated lipids showed an effect of time during the postprandial period (*q* < 0.05).

**Figure 1:**
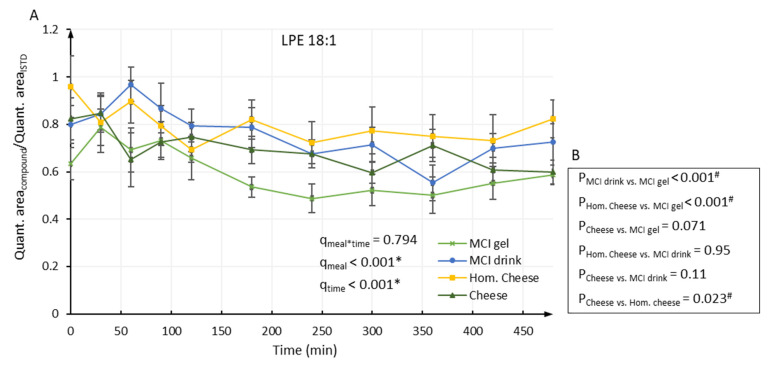
(**A**) Relative level of LPE 18:1 in plasma samples. Data were analyzed by repeated measures using a linear mixed model. Overall participant and within-visit participant differences were included as random factors and the analyses were adjusted for BMI, age and visit. *q*-values indicate FDR-adjusted *p*-values for meal–time interactions (q_meal*time_), the effect of meal (q_meal_) and the effect of time (q_time_). * indicate significant differences (*q* < 0.05). (**B**) *p*-values obtained from Tukey’s all-pairwise comparison conducted when a significant meal effect was observed in repeated measures analysis. ^#^ indicate significant differences (*p* < 0.05).

**Figure 2:**
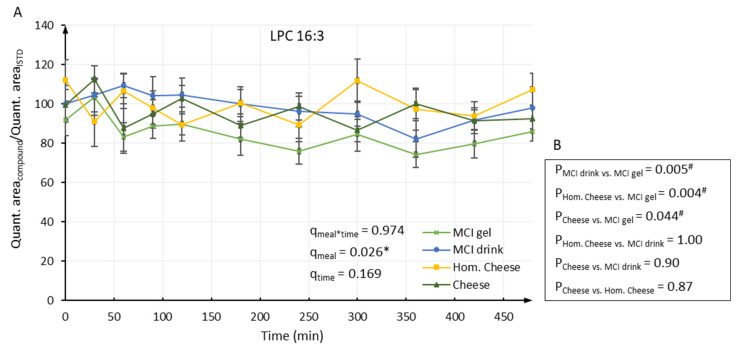
(**A**) Relative level of LPC 16:3 in plasma samples. Data were analyzed by repeated measures using a linear mixed model. Overall participant and within-visit participant differences were included as random factors and the analyses were adjusted for BMI, age and visit. *q*-values indicate FDR-adjusted *p*-values for meal–time interactions (q_meal * time_), the effect of meal (q_meal_) and the effect of time (q_time_). * indicate significant differences (*q* < 0.05). (**B**) *p*-values obtained from Tukey’s all-pairwise comparison conducted when a significant meal effect was observed in repeated measures analysis. ^#^ indicate significant differences (*p* < 0.05).

For the nine phospholipids showing an effect of meal, differences between the treatment groups were evaluated by Tukey’s all-pairwise comparison. For all the nine phospholipids, significant differences were found between consumption of MCI Drink and MCI Gel and between consumption of Hom. Cheese and MCI Gel. Generally, consumption of MCI Gel appeared to result in the lowest postprandial level of the nine phospholipids and, for all of the nine phospholipids, a significant difference was found between consumption of MCI Gel and MCI Drink and between consumption of MCI Gel and Hom. Cheese (Figure 1, Figure 2 and Figure S3). For LPE 18:2 and LPC 16:3, significant differences were also found between consumption of cheese and MCI Gel (Figures S3.1 and S3.2). Likewise, for LPE 18:1 and LPE 20:2, significant differences were found between consumption of cheese and Hom. Cheese (Figure 1 and Figure S3.4).

## 3. Discussion

In the present study, no meal–time interactions were revealed for any of the analysed phospholipids. However, lower phospholipid levels were found after consumption of the MCI gel compared to the MCI drink and the Hom. cheese in nine of the 53 investigated phospholipids.

Increasing evidence indicates that not only the single nutrients, but also the complex structure of a given food can influence its health effects following consumption. Thus, the nature of the food matrix may play an important role by affecting the absorption and digestion of a given food [7]. In fact, previous studies found that the dairy matrix can affect the postprandial response and nutritional properties of dairy products [6,22]. Milk contains phospholipids, which are located within the MFGM, the trilayered membrane surrounding the fat globules. Dairy phospholipids play an important role in the emulsification of fat in milk, but their impact on human health has also received attention [23,24]. Hence, studies have indicated potential health beneficial effects of dietary phospholipid consumption including decreased cholesterol absorption, suppressed intestinal inflammation, and modulation of the gut microbiota [14,23,25,26]. Previous studies have shown that the postprandial blood phospholipid status in humans can be affected by diet [27,28], and a recent study also revealed that the postprandial changes in blood phospholipid are perturbed in people with metabolic syndrome [29]. Meikle et al. (2015) investigated the consumption of dairy fat and soy oil and found that the source of dietary fat affected the postprandial plasma phospholipid concentrations in men [28]. Thus, a few studies have investigated the effect of dietary sources on postprandial blood phospholipid status, but little is known about the effect of the dairy matrix on postprandial blood phospholipids levels. In the present study, the majority of the 53 phospholipids examined in plasma showed minor concentration changes during the postprandial period and some remained steady in level. For the majority of the analysed phospholipids in the present study, no meal effect was found. These results are in accordance with those of Weiland et al. (2016), who found that milk phospholipid consumption did not affect the overall plasma phospholipid levels [30]. However, in the present study, for nine of the 53 investigated phospholipids, an effect of meal was observed. Thus, a meal effect was found for six out of eight measured LPEs and three out of seven measured LPCs. Generally, for these nine phospholipids, lower levels were found after consumption of the MCI Gel compared to the MCI Drink and the Hom. Cheese. Meikle et al. (2015) also found that consumption of a dairy meal differently affected postprandial lysophospholipid levels. Thus, dairy meal consumption increased postprandial levels of total LPEs and lysophosphatidylinositol, whereas no changes were observed for postprandial levels of total LPC [28]. When consumed, phospholipids are hydrolyzed in the intestine, resulting in the formation of lysophospholipids and free fatty acids [31]. Hence, in the present study, the metabolism of specific phospholipids might have been differently affected, leading to different effects on specific LPEs and LPCs.

The observed meal effects were not reflected in the phospholipid content of the analysed meals, which did not differ between the meals (data not shown). The lower level of phospholipids following consumption of the MCI Gel might be ascribed to a number of different factors. Comparing the MCI Gel and the MCI Drink, the MCI Gel had an increased viscosity prior to digestion [21]. Previous studies have indicated that an increased viscosity can slow gastric emptying rate [32,33], which could possibly explain the lower level of phospholipids in plasma following the MCI Gel. In addition, the size of the fat droplets in the dairy products might play a role in postprandial phospholipid response in the present study. Analyses of the dairy products revealed that the MCI Gel had a significantly larger fat droplet size compared to the MCI Drink and Hom. Cheese prior to digestion [21]. In fact, it has previously been found that larger fat droplets sizes can result in a slower digestion [34]. However, during in vitro digestion, the particle size of the products changed. The MCI Gel had the largest volume D(4.3) weighted diameter values during the gastric digestion, while during the intestinal digestion, the D(4.3) increased for the Cheese to a level significantly (*p* < 0.01) larger than for the three other products [21]. For surface D(3.2) weighed diameters, on the other hand, Hom. Cheese had the significantly highest level during gastric digestion, and again the Cheese was shown to have the largest particle sizes in the intestinal digestion phase [21]. In addition to fat droplet size, it could be speculated that the stability and intactness of the MFGM may also affect the postprandial phospholipid status. Under physiological pH and temperature, the MFGM is regarded as relatively stable, but various factors including homogenization and changes in temperature, salt and pH can induce destabilization [35]. Therefore, since the majority of the milk phospholipids are expected to be located within the MFGM, variations in the mentioned factors among the dairy products could potentially play a role in the observed meal effects. However, further studies are required to identify possible effects of the stability of the MGFM on phospholipid absorption.

The present study has some limitations. As a normalization of the LC-MS data was necessary to overcome between-batch variations resulting from drifts during data acquisition, relative concentrations of phospholipids are reported. While this quantitation allows a comparison of different meals, subjects etc., a direct comparison of the reported concentrations to other studies is not possible. In terms of mechanistic data interpretation, the phospholipid analyses were based on unfractionated blood plasma. Fractionation into different lipoproteins and chylomicrons could be expected to provide additional information in relation to the exogenous or endogenous origin of the phospholipids.

Differences in the amount of phospholipids present in the meals may impact the post prandial phospholipid profile, and another limitation with the present study is the fact that the amount of phospholipids in the meals was not quantified. Neither was it characterized how the processing, i.e., the homogenization of the cheese, affected the phospholipid composition. However, our main goal was to examine differences in the postprandial kinetics as functions of the different meal products’ textures and structures.

In conclusion, no meal–time interactions were revealed; however, for nine of the 53 phospholipids, a meal effect was found. The results indicated a lower plasma level of specific LPEs and LPCs following consumption of the MCI Gel compared to the MCI Drink and Hom. Cheese. However, further studies are needed in order to reveal the detailed effect of the dairy matrix on postprandial phospholipids.

## 4. Materials and Methods

### 4.1. Dairy Products

The dairy products differed in structural (solid, semi-solid, and liquid) and textural integrity. Briefly, a commercial full-fat cheddar cheese (Cheese) (Arla Foods Ltd., Leeds, UK) with native milk fat globules and an intact protein network constituted the solid cheese matrix. A semi-solid cheese matrix with homogenized milk fat globules and partial loss of the protein network (Hom. Cheese) was produced from the commercial full-fat cheese through homogenization. A solution of micellar casein isolate (MCI) with cream and salt (MCI Drink), containing native milk fat globules but void of a protein network, constituted the liquid cheese matrix. Finally, a gel was produced based on the MCI Drink mixed with glucono-δ-lactone to form a semi-solid matrix (MCI Gel) with a gelled protein network and native milk fat globules. In order to obtain similar macro and micro nutrient content and ensure an overall energy consumption, the dairy products were served with bread and water [20].

### 4.2. Test Participants and Study Design

**Figure 3:**
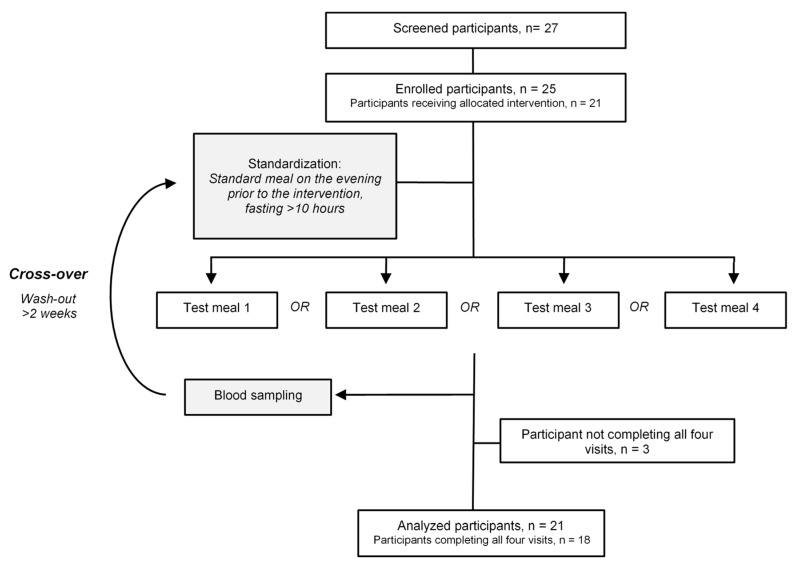
Study design.

The study was an acute, cross-over intervention study consisting of four test days (Figure 3) conducted from September 2018 to March 2019 at the Department of Nutrition, Exercise and Sports, University of Copenhagen, Denmark. Co-primary outcomes (postprandrial triglycerides and Apolipoprotein B-48) are reported in Kjølbæk et al. [20] and in the present study, data from the analysis of postprandial plasma phospholipids are reported. Participants were recruited using announcements in Danish newspapers and various internet platforms, and a total of 25 men were enrolled. The inclusion criteria were as follows: healthy men (between 18 and 40 years), non-smoker, body mass index (BMI): 18.5–24.9 kg/m^2^, hemoglobin concentration ≥8.4 mmol/L. Participants meeting the exclusion criteria were omitted [20]. Participants who completed a minimum of two test days (*n* = 21) were included in the analyses, and 18 participants completed all four test days. For standardization, the study participants were instructed to avoid alcohol, medication and strenuous physical activity 48 h prior to the test day. Study participants consumed a standardized meal on the evening prior to the test day and were instructed to finish the meal before 10 in the evening, after which they had to fast until receiving the test meal the following day. For all participants, the order in which they received the four test meals was randomized. On each test day, participants were weighed upon arrival and fasting blood samples were drawn (time 0 min.), after which the participants received a test meal (4.7 MJ) that included one of the four dairy products, and 1.5 g paracetamol, which was to be consumed within 15 min. During the 8 h period, participants drank two glasses of water. Postprandial blood samples were drawn at time points 30, 60, 90, 120, 180, 240, 300, 360, 420 and 480 min. and plasma was collected and stored at −80 °C until analysis.

### 4.3. Materials

The lipid standards 3-sn-phosphatidylethanolamine (from bovine brain, ≥98%) and L-α-phosphatidylcholine (from egg yolk, ≥99%) were purchased from Merck KGaA (Darmstadt, Germany), sphingomyelin (from bovine buttermilk, >98%), lyso-phosphatidylethanolamine (from bovine brain, >98%) and Ceramide (mixture, >98%) were purchased from Larodan (Solna, Sweden), and L-α-Lysophosphatidylcholine (from egg yolk, >98%) was purchased from Santa Cruz Biotechnology (Dallas, TX, USA). The deuterated internal standard SPLASH^®^ LIPIDOMIX^®^ Mass Spec Standard (Avanti Polar Lipids, Alabaster, AL, USA) was purchased from Merck KGaA (Darmstadt, Germany). Methanol (HiPerSolv CHROMANORM^®^, LC-MS grade, ≥99.9%), acetonitrile (HiPerSolv CHROMANORM^®^, LC-MS grade, ≥99.9%) and water (HiPerSolv CHROMANORM^®^ LC-MS grade) were purchased from VWR (Søborg, Denmark), and chloroform (≥99.9%, LC-MS grade) was purchased from Rathburn Chemicals Ltd. (Walkerburn, Scotland). Ammonium formate (LC-MS grade, 99.0%) was purchased from Sigma-Aldrich (St. Louis, MO, USA), and formic acid (LC-MS LiChropur^®^, 98–100%) was purchased from Merck KGaA (Darmstadt, Germany). A water purification system (Holm & Halby, Brøndby, Denmark) was used to prepare ultrapure (18.2 MΩ) water.

### 4.4. Extraction of Plasma Lipids

Plasma samples (*n* = 880) were randomized prior to extraction. Extraction of polar lipids was based on a modified version of the Bligh and Dyer method [36]. Samples were thawed on ice and whirl pooled, after which 100 µL plasma was transferred to a centrifuge tube. To the centrifuge tube, 1 µL deuterated internal standard was added and briefly mixed on a vortex mixer, followed by the addition of 737 µL ice cold methanol:water solution (*v*/*v* 2:0.8). The samples were briefly mixed on a vortex mixer, after which 263 µL ice cold chloroform was added and followed by vortex mixing. After addition of the extraction solvents, the centrifuge tubes were centrifuged at 20,000× *g* for 10 min at 4 °C and the organic and aqueous phases were transferred to a new centrifuge tube, discarding the interphase. Solvents were vaporized at 30 °C under vacuum, and the resulting lipid extracts were suspended in 1000 µL ice cold methanol. Finally, the samples were centrifuged at 20,000× *g* for 10 min at 4 °C and the supernatants were transferred to new centrifuge tubes and stored at −80 °C until analysis. Prior to analysis, the samples were further centrifuged (20,000× *g* for 10 min at 4 °C) to remove insoluble particles and the supernatant transferred to 96 well plates.

### 4.5. Extraction of Dairy Lipids

For preparation of the dairy products, samples (*n* = 4) were prepared in technical triplicates. The samples were frozen at −18 °C and lyophilized. After lyophilization, samples were transferred to a mortar and ground. Liquid nitrogen was added to the mortar to ensure homogenous samples. To a centrifuge tube, 20 mg sample was transferred and lipids were extracted using the same method as for the plasma samples.

### 4.6. Standards and Calibration Curves

Stock solutions of polar lipid standards, one for each of the six lipid classes, were prepared in chloroform (phosphatidylcholine (PC), phosphatidylethanolamine (PE), lysophosphatidylcholine (LPC), ceramide (CER) and sphingomyelin (SM): 2.5 g/L, lysophosphatidylethanolamine (LPE): 1 g/L). For calibration, different concentration levels were constructed by dilution of the stock solutions in methanol (PC: 6.7 × 10^−6^–1 g/L, PE: 3.4 × 10^−6^–0.5 g/L, LPC and SM: 2.7–0.4 g/L, LPE: 1.1 × 10^−7^–1.6 × 10^−2^ g/L, Cer: 6.7 × 10^−7^–0.1 g/L). Deuterated internal standard (1 µL) was added to each calibration level. A total of three deuterated internal standards were used; deuPC was used to quantitate PC and PE, deuLPC was used for LPC and LPE, and deuSM was used for SM and Cer. To monitor batch variation, two concentration levels of mixed lipid standards in methanol were used for the quality controls (QC). Stock solutions, calibration levels and QCs were stored in glass tubes at −20 °C.

### 4.7. Liquid Chromatography Tandem Mass Spectrometry

The method for semi-quantitative analysis of polar lipids (ng/µL) was based on a LC-ESI-MS/MS method as previously described [19], with few modifications. Briefly, the LC-MS system consisted of an Agilent 1290 Ultra Performance Liquid Chromatography coupled to an Agilent 6495 Triple Quadrupole Mass Spectrometer (Agilent Technologies, Palo Alto, CA, USA) operated in ESI positive mode. Eluent A consisted of water with 10 mM ammonium formate (adjusted to pH 3) and eluent B consisted of acetonitrile. Ninety-six-well plates containing the extracted samples were placed in the multisampler at 4 °C and 5 µL was injected onto an ACQUITY UPLC BEH HILIC Column (1.7 µm, 2.1 × 100 mm) coupled with an ACQUITY UPLC BEH HILIC VanGuard Pre-column (1.7 µm, 2.1 mm × 5 mm, Waters Corporation, Milford, MA, USA). A flowrate of 0.500 mL/min and column temperature of 25 °C was used. The percentage of eluent B used for chromatographic separation was as follows: 0.00–0.10 min: 95%; 0.10–20 min: 95–80%; 20–21 min: 80–70%; 21–23 min: 70%; 23–24 min: 70–95%; 24–26 min: 95% (column equilibration). After 15 min, all compounds of interest were eluted; therefore, for the remainder of the analytic run, the eluate was diverged from MS to waste. The ESI source was operated in positive mode and source gas temperature was set to 120 °C, and gas flow to 11 L/min. The nebulizer pressure was set to 35 psi, and the temperature and sheath gas flow was set to 350 °C and 10 L/min, respectively. The capillary voltage was set to 4000 V and the nozzle voltage to 500 V. The dual ion funnel system was set to 210 V and 160 V, for the high and low-pressure funnel, respectively. Each batch was divided into analytical blocks, consisting of six samples (a blank was injected after the third sample), flanked by a QC low and QC high before and after injection of the samples. Moreover, two blanks were injected in between blocks as well as before and after injection of QCs to avoid carry-over. For semi-quantification of lipids, a dynamic multiple reaction monitoring MS method and a mix of deuterated (deu) PC, LPC and SM internal standards was used; deuPC was used to quantitate PC and PE, whereas deuLPC was used for LPC and LPE, and deuSM was used for SM and Cer. The MS method that was used enables identification at the lipid species level but does not reveal the stereo specific numbering of the fatty acyl residues of the carbon backbone. Thus, the annotation of a glycerophospholipid containing two fatty acyl residues with, e.g., chain lengths of 18 carbons and two double bonds and 18 carbons and one double bond, is written 18:2_18:1, in accordance with the LIPID MAPS consortium [37] nomenclature.

### 4.8. LC-MS Data Preprocessing

LC-MS data processing was performed using the Agilent MassHunter Workstation Software—Data Acquisition for 6400 Series Triple Quadrupole program version B.07.00. A total of 53 phospholipids were annotated. A complete list of the investigated phospholipids is provided in Supplementary Material Table S5. All 1307 plasma samples were analyzed by LC-MS in 41 batches. Each batch included 25–30 real samples, six high concentration quality control mixture samples (QC), and six low concentration QC samples. Both QC samples, high QC and low QC, were run in between six real samples within each batch. While within-batch variations of the QC samples were insignificant, larger between-batch variations in the data were observed. Therefore, the LC-MS data were corrected for the between-batch effect using the high QC samples run within batches. The following equation was used for the batch correction:

X_COR(*i*,*j*,*b*)_ = (X_RAW(*i*,*j*,*b*)_/mean(QC_(:,*j*,*b*)_)) × mean(QC_(:,*j*,*B*)_) (1)

where, X_COR_ is between-batch effect corrected data, X_RAW_ is a raw data, QC is high concentration quality control mixture sample, *i* = sample, *j* = variable, *b* = batch, *B* = all batches.

According to Equation (1), a peak area of each phospholipid was normalized (divided) by the mean area of the corresponding internal standard analyzed from high QC samples run within the same batch. Then, in order to correct for the magnitude change, normalized peak areas were multiplied by the mean of the corresponding internal standard analyzed from high QC samples run across all batches.

### 4.9. Statistical Analysis

Repeated measurements using a linear mixed model were conducted to investigate meal–time interactions. Overall participant and within-visit participant differences were included as random factors and the analyses were adjusted for BMI, age and visit. Normal probability plots and residuals plots were used to test the assumptions of normal distribution and homogeneity of variance. If the assumptions were not met, data were log-transformed. In order to control for multiple testing, *p*-values were adjusted by the false discovery rate (FDR) using the Benjamini and Hochberg approach [38]. FDR-adjusted *p*-values are denominated *q*-values and a *q*-value < 0.05 was considered significant. When a meal effect was found (*q* < 0.05), differences between the treatment groups were evaluated by Tukey’s all-pairwise comparison. One-way ANOVA at time 0 min. revealed no significant differences among treatment groups at the beginning of the postprandial period. All statistical analyses were conducted using the statistical software RStudio (version 1.3.1093).

## Figures and Tables

**Table 1:** Relative level of lysophosphatidylethanolamine (LPE) in plasma samples.* Data were analyzed by repeated measures using a linear mixed model. Overall participant and within-visit participant differences were included as random factors and the analyses were adjusted for BMI, age and visit. *q*-values indicate FDR-adjusted *p*-values for meal–time interactions (q_meal * time_), the effect of meal (q_meal_) and the effect of time (q_time_). * indicate significant differences (*q* < 0.05).

Time (min)	0	30	60	90	120	180	240	300	360	420	480	q_meal * time_	q_meal_	q_time_
**LPE 16:0**														
Cheese	0.018	0.018	0.015	0.015	0.017	0.016	0.017	0.014	0.017	0.015	0.014	0.79	0.29	0.11
Hom. Cheese	0.026	0.021	0.019	0.018	0.017	0.021	0.018	0.024	0.018	0.021	0.019			
MCI Drink	0.017	0.020	0.023	0.019	0.017	0.018	0.016	0.019	0.015	0.018	0.016			
MCI Gel	0.015	0.019	0.016	0.017	0.018	0.014	0.014	0.016	0.016	0.015	0.014			
**LPE 18:2**														
Cheese	0.335	0.412	0.303	0.295	0.317	0.309	0.296	0.281	0.314	0.286	0.332	0.69	0.001 *	0.30
Hom. Cheese	0.419	0.278	0.367	0.307	0.282	0.308	0.283	0.316	0.354	0.300	0.358			
MCI Drink	0.298	0.319	0.398	0.358	0.330	0.404	0.302	0.313	0.243	0.271	0.308			
MCI Gel	0.263	0.288	0.279	0.290	0.286	0.246	0.251	0.250	0.224	0.268	0.268			
**LPE 18:1**														
Cheese	0.824	0.846	0.650	0.726	0.747	0.692	0.675	0.596	0.711	0.607	0.598	0.79	<0.001 *	<0.001 *
Hom. Cheese	0.959	0.807	0.896	0.795	0.694	0.820	0.722	0.774	0.750	0.730	0.823			
MCI Drink	0.800	0.845	0.969	0.868	0.794	0.787	0.675	0.715	0.553	0.698	0.727			
MCI Gel	0.633	0.787	0.692	0.731	0.659	0.535	0.488	0.522	0.501	0.553	0.587			
**LPE 18:0**														
Cheese	1.780	1.878	1.498	1.636	1.742	1.615	1.564	1.361	1.642	1.371	1.329	0.79	0.026 *	0.001 *
Hom. Cheese	2.141	1.723	1.923	1.674	1.613	1.838	1.622	1.819	1.733	1.638	1.814			
MCI Drink	1.714	1.856	2.139	1.953	1.793	1.762	1.561	1.665	1.256	1.520	1.572			
MCI Gel	1.499	1.792	1.590	1.690	1.641	1.347	1.254	1.339	1.229	1.329	1.365			
**LPE 20:3**														
Cheese	0.105	0.121	0.091	0.102	0.096	0.097	0.095	0.105	0.116	0.098	0.120	0.79	0.016 *	0.010 *
Hom. Cheese	0.126	0.093	0.129	0.107	0.090	0.103	0.099	0.107	0.119	0.113	0.137			
MCI Drink	0.111	0.106	0.122	0.107	0.103	0.111	0.108	0.109	0.090	0.116	0.124			
MCI Gel	0.090	0.093	0.090	0.104	0.094	0.078	0.083	0.090	0.085	0.106	0.104			
**LPE 20:2**														
Cheese	0.103	0.109	0.077	0.089	0.085	0.085	0.082	0.070	0.088	0.073	0.075	0.79	<0.001 *	<0.001 *
Hom. Cheese	0.124	0.102	0.108	0.095	0.086	0.096	0.087	0.100	0.092	0.090	0.105			
MCI Drink	0.098	0.109	0.116	0.108	0.096	0.092	0.081	0.083	0.062	0.091	0.095			
MCI Gel	0.076	0.094	0.080	0.091	0.080	0.061	0.056	0.067	0.063	0.073	0.075			
**LPE 20:1**														
Cheese	1.332	1.384	1.015	1.121	1.188	1.077	1.034	0.968	1.197	1.019	1.057	0.79	0.001 *	<0.001 *
Hom. Cheese	1.533	1.277	1.353	1.165	1.120	1.201	1.117	1.167	1.228	1.163	1.370			
MCI Drink	1.266	1.393	1.497	1.385	1.215	1.245	1.052	1.206	0.891	1.190	1.264			
MCI Gel	1.066	1.222	1.027	1.163	1.036	0.819	0.795	0.866	0.830	0.972	1.024			
**LPE 22:6**														
Cheese	1.108	1.227	0.962	1.017	1.130	0.967	1.063	0.952	1.140	0.984	1.038	0.79	0.07	0.30
Hom. Cheese	1.295	1.011	1.155	1.120	0.982	1.110	1.014	1.246	1.096	1.106	1.204			
MCI Drink	1.166	1.183	1.279	1.187	1.190	1.175	1.106	1.088	0.979	1.080	1.165			
MCI Gel	1.033	1.148	0.966	1.024	0.979	0.949	0.904	0.991	0.842	0.946	0.955			

* Fatty acid moiety is shown with number of carbons followed by the number of double bond of carbons in the fatty acid chain.

**Table 2:** Relative level of lysophosphatidylcholine (LPC) in plasma samples.* Data were analyzed by repeated measures using a linear mixed model. Overall participant and within-visit participant differences were included as random factors and the analyses were adjusted for BMI, age and visit. *q*-values indicate FDR-adjusted *p*-values for meal–time interactions (q_meal * time_), the effect of meal (q_meal_) and the effect of time (q_time_). * indicate significant differences (*q* < 0.05).

Time (min)	0	30	60	90	120	180	240	300	360	420	480	q_meal * time_	q_meal_	q_time_
**LPC 16:3**														
Cheese	99.428	112.408	87.591	94.724	102.710	88.845	98.695	86.458	99.880	91.332	92.618	0.79	0.026 *	0.17
Hom. Cheese	111.998	90.966	106.467	97.927	89.453	100.357	89.250	111.806	97.412	93.725	107.311			
MCI Drink	99.916	104.535	109.441	104.144	104.554	99.942	96.144	94.973	82.139	91.793	97.931			
MCI Gel	91.769	103.383	83.215	88.737	89.650	82.000	75.879	84.615	74.239	79.843	86.073			
**LPC 18:4**														
Cheese	0.192	0.265	0.172	0.185	0.196	0.186	0.269	0.175	0.240	0.285	0.207	0.69	0.56	0.031 *
Hom. Cheese	0.240	0.150	0.133	0.202	0.244	0.223	0.194	0.294	0.211	0.247	0.295			
MCI Drink	0.225	0.238	0.222	0.222	0.229	0.155	0.254	0.198	0.178	0.245	0.265			
MCI Gel	0.185	0.242	0.141	0.166	0.164	0.186	0.168	0.253	0.206	0.200	0.223			
**LPC 18:1**														
Cheese	37.100	40.785	31.934	32.954	34.604	31.160	35.798	32.833	40.197	37.388	39.552	0.79	0.038 *	0.005 *
Hom. Cheese	43.961	32.096	39.357	34.708	33.136	36.201	33.256	39.231	39.143	39.298	46.104			
MCI Drink	37.529	38.037	40.413	38.786	37.626	38.473	35.659	37.146	32.123	40.174	42.776			
MCI Gel	32.524	35.282	30.447	33.027	31.176	29.543	27.902	32.784	29.748	35.714	36.609			
**LPC 18:0**														
Cheese	42.735	47.521	36.862	39.382	43.097	37.804	41.202	37.245	43.150	38.534	40.294	0.79	0.045 *	0.30
Hom. Cheese	50.650	39.148	45.818	41.800	38.243	44.158	39.142	46.650	42.214	43.289	46.385			
MCI Drink	45.162	45.427	49.535	46.238	45.999	47.192	42.386	43.521	37.251	41.634	44.870			
MCI Gel	39.870	43.515	37.074	39.537	39.408	36.150	35.096	37.522	32.041	36.311	37.531			
**LPC 20:0**														
Cheese	0.436	0.388	0.489	0.560	0.883	0.606	0.670	0.632	0.751	0.602	0.762	0.91	0.80	0.049 *
Hom. Cheese	0.516	0.426	0.544	0.572	0.576	0.760	0.732	0.583	0.679	0.763	0.655			
MCI Drink	0.442	0.418	0.550	0.463	0.569	0.897	0.502	0.722	0.588	0.671	0.633			
MCI Gel	0.371	0.399	0.664	0.677	0.622	0.508	0.543	0.556	0.653	0.507	0.562			
**LPC 22:0**														
Cheese	0.00014	0.00008	0.00007	0.00007	0.00008	0.00007	0.00006	0.00007	0.00013	0.00008	0.00010	0.83	0.45	0.48
Hom. Cheese	0.00008	0.00008	0.00007	0.00004	0.00005	0.00009	0.00006	0.00007	0.00009	0.00008	0.00011			
MCI Drink	0.00007	0.00006	0.00009	0.00006	0.00009	0.00009	0.00007	0.00006	0.00008	0.00006	0.00009			
MCI Gel	0.00005	0.00004	0.00007	0.00011	0.00007	0.00007	0.00008	0.00008	0.00007	0.00005	0.00006			
**LPC 24:0**														
Cheese	0.586	0.539	0.647	0.713	0.932	0.613	0.501	0.958	0.636	0.636	0.772	0.89	0.76	0.48
Hom. Cheese	0.851	0.627	0.898	0.964	0.504	0.666	0.553	0.571	0.625	0.626	0.808			
MCI Drink	0.793	0.561	0.730	0.695	0.883	0.803	0.729	0.832	0.753	0.683	0.824			
MCI Gel	0.561	0.533	0.838	0.701	0.592	0.556	0.719	0.411	0.582	0.626	0.533			

* Fatty acid moiety is shown with number of carbons followed by the number of double bond of carbons in the fatty acid chain.

## Data Availability

Pseudo-anonymized data can be made available upon request before 2029 via a data sharing contract. From 2029, fully anonymized data can be transferred.

## Supplementary Materials

The following are available online at https://www.mdpi.com/article/10.3390/metabo11070454/s1, Table S1: Relative level of ceramide (CER) in plasma samples, Table S2: Relative level of sphingomyelin (SM) in plasma samples, Table S3: Relative level of phosphatidylethanolamine (PE) in plasma samples, Table S4: Relative level of phosphatidylcholine (PC) in plasma samples, Table S5: List of investigated phospholipids, Figure S1: PCA Scores plot, Figure S2: PCA loadings plot, Figure S3: Relative level of phospholipids in plasma samples for (1) LPE 18:2, (2) LPE 18:0, (3) LPE 20:3, (4) LPE 20:2, (5) LPE 20:1, (6) LPC 18:1, (7) LPC 18:0.
