# Supplementary material for: Progression of Postprandial Blood Plasma Phospholipids Following Acute Intake of Different Dairy Matrices: A Randomized Crossover Trial
Source: Metabolites. 2021 Jul 14;11(7):454. doi: 10.3390/metabo11070454 (PMC8307057; doi:10.3390/metabo11070454)
Supplement: Supplementary file 1 [file metabolites-11-00454-s001.zip › metabolites-1267395-supple/Dairymat_Supplementary material_Metabolites_Revised.pdf]

Supplementary data

Table S1. Relative level of ceramide (CER) in plasma samples. Data were analyzed by repeated measures using linear mixed model. Overall participant and within-visit participant differences were included as random factors and the analyses were adjusted for BMI, age and visit. q-values indicate FDR-adjusted p-values for meal-time interactions ($q_{meal*time}$), the effect of meal (q_{meal}) and the effect of time (q_{time}).

	0	30	60	90	120	180	240	300	360	420	480	$q_{meal*time}$	q_{meal}	q_{time}
**CER 18:0_18:2**														
Cheese	0.003	0.003	0.001	0.001	0.001	0.001	0.001	0.002	0.001	0.001	0.001	0.833	0.562	0.392
Hom. Cheese	0.001	0.002	0.002	0.001	0.001	0.001	0.001	0.001	0.013	0.001	0.001			
MCI Drink	0.014	0.009	0.001	0.001	0.001	0.004	0.001	0.002	0.013	0.009	0.004			
MCI Gel	0.001	0.001	0.001	0.001	0.001	0.001	0.001	0.001	0.001	0.001	0.001			
**CER 18:0_18:1**														
Cheese	0.007	0.009	0.007	0.006	0.012	0.005	0.006	0.010	0.009	0.007	0.014	0.687	0.562	0.235
Hom. Cheese	0.008	0.007	0.005	0.006	0.008	0.006	0.008	0.006	0.014	0.008	0.009			
MCI Drink	0.018	0.011	0.008	0.005	0.005	0.008	0.007	0.007	0.010	0.009	0.010			
MCI Gel	0.006	0.008	0.009	0.008	0.008	0.007	0.009	0.009	0.009	0.007	0.008			
**CER 18:0_20:2**														
Cheese	0.001	0.001	0.001	0.001	0.002	0.001	0.001	0.001	0.001	0.001	0.001	0.794	0.791	0.713
Hom. Cheese	0.001	0.001	0.001	0.001	0.001	0.001	0.001	0.001	0.002	0.001	0.002			
MCI Drink	0.002	0.002	0.001	0.001	0.001	0.001	0.001	0.001	0.002	0.001	0.001			
MCI Gel	0.001	0.001	0.001	0.001	0.001	0.001	0.001	0.001	0.001	0.001	0.001			
**CER 18:0_22:1**														
Cheese	0.116	0.151	0.125	0.126	0.236	0.098	0.121	0.159	0.173	0.112	0.195	0.794	0.695	0.479
Hom. Cheese	0.152	0.123	0.104	0.128	0.146	0.132	0.162	0.125	0.172	0.165	0.156			
MCI Drink	0.285	0.217	0.147	0.109	0.108	0.150	0.129	0.160	0.134	0.153	0.214			
MCI Gel	0.124	0.151	0.142	0.146	0.157	0.138	0.148	0.159	0.161	0.127	0.114			
**CER 18:0_24:0**														
Cheese	0.095	0.134	0.096	0.101	0.183	0.081	0.097	0.129	0.141	0.099	0.156	0.794	0.564	0.170
Hom. Cheese	0.132	0.107	0.092	0.101	0.121	0.108	0.127	0.101	0.146	0.137	0.131			
MCI Drink	0.307	0.189	0.121	0.099	0.087	0.119	0.102	0.133	0.118	0.134	0.205			
MCI Gel	0.095	0.113	0.110	0.116	0.119	0.106	0.116	0.138	0.128	0.101	0.097			
**CER 18:0_26:3**														
Cheese	0.003	0.003	0.002	0.002	0.004	0.001	0.002	0.002	0.003	0.002	0.003	0.794	0.564	0.764
Hom. Cheese	0.003	0.002	0.002	0.002	0.003	0.002	0.003	0.002	0.005	0.004	0.002			
MCI Drink	0.009	0.005	0.003	0.002	0.002	0.004	0.002	0.003	0.006	0.004	0.003			

MCI Gel	0.002	0.002	0.002	0.003	0.003	0.003	0.003	0.002	0.003	0.002	0.002			
**CER 18:0_26:2**														
Cheese	0.005	0.005	0.004	0.004	0.006	0.004	0.005	0.005	0.005	0.005	0.007	0.794	0.149	0.630
Hom. Cheese	0.005	0.005	0.004	0.004	0.005	0.004	0.005	0.003	0.007	0.006	0.005			
MCI Drink	0.010	0.009	0.005	0.004	0.003	0.006	0.004	0.005	0.005	0.007	0.006			
MCI Gel	0.004	0.004	0.004	0.005	0.005	0.004	0.005	0.004	0.004	0.004	0.004			

*Table S2. Relative level of sphingomyelin (SM) in plasma samples. Data were analyzed by repeated measures using linear mixed model. Overall participant and within-visit participant differences were included as random factors and the analyses were adjusted for BMI, age and visit. q-values indicate FDR-adjusted p-values for meal-time interactions ($q_{meal*time}$), the effect of meal (q_{meal}) and the effect of time (q_{time}). * indicate significant differences (q < 0.05).*

	0	30	60	90	120	180	240	300	360	420	480	$q_{meal*time}$	q_{meal}	q_{time}
**SM 18:0_14:0**														
Cheese	1.126	1.541	1.095	0.995	1.470	1.137	1.440	1.261	1.358	1.502	1.442	0.902	0.823	0.001*
Hom. Cheese	1.403	1.253	1.252	1.146	1.093	1.203	1.310	1.544	1.621	1.559	1.512			
MCI Drink	1.212	1.687	1.275	1.250	1.397	1.326	1.704	1.380	1.237	1.260	1.493			
MCI Gel	1.099	1.251	1.120	1.141	1.158	1.075	1.286	2.004	1.538	1.389	1.590			
**SM 18:0_20:0**														
Cheese	5.226	6.038	5.214	4.552	5.543	5.038	5.473	5.676	6.041	5.707	6.141	0.833	0.564	0.143
Hom. Cheese	5.827	4.734	5.654	4.927	4.959	5.054	4.946	6.209	5.494	5.713	6.030			
MCI Drink	4.875	5.529	5.654	5.209	5.213	6.107	5.187	5.977	4.980	5.480	5.379			
MCI Gel	5.049	5.418	4.934	5.001	5.465	4.794	4.936	5.370	5.016	5.477	5.754			
**SM 18:0_22:1**														
Cheese	15.521	17.013	15.465	14.489	17.481	14.973	15.927	17.488	19.044	16.724	18.949	0.833	0.564	0.235
Hom. Cheese	17.638	14.876	17.551	15.818	14.388	15.108	15.513	18.510	17.211	18.322	18.512			
MCI Drink	15.615	16.086	18.206	16.681	16.690	19.253	16.708	19.475	15.764	17.480	17.154			
MCI Gel	15.438	15.567	14.952	15.922	17.381	14.654	15.799	15.742	15.447	17.386	17.613			
**SM 18:1_22:0**														
Cheese	15.537	17.016	15.574	14.282	17.430	15.141	15.952	17.474	19.030	16.749	19.134	0.833	0.564	0.175
Hom. Cheese	17.614	14.919	17.475	15.587	14.440	15.058	15.552	18.503	17.091	18.264	18.537			
MCI Drink	15.504	16.041	18.218	16.603	16.746	19.468	16.604	19.496	15.732	17.525	17.148			
MCI Gel	15.433	15.538	14.864	15.779	17.443	14.534	16.023	15.736	15.589	17.456	17.685			
**SM 18:0_22:0**														
Cheese	0.891	0.978	0.835	0.833	0.970	0.820	0.876	0.966	1.037	0.944	1.064	0.833	0.564	0.143
Hom. Cheese	0.910	0.787	0.975	0.906	0.764	0.866	0.771	1.047	0.877	0.984	1.027			
MCI Drink	0.836	0.925	0.918	0.842	0.883	1.108	0.936	1.167	0.911	0.985	0.971			
MCI Gel	0.862	0.900	0.813	0.886	0.997	0.793	0.866	0.914	0.795	0.946	0.972			
**SM 18:1_23:0**														
Cheese	3.288	3.615	2.913	2.845	3.346	3.012	3.353	3.176	3.575	3.213	3.478	0.794	0.564	0.677
Hom. Cheese	3.836	2.844	3.453	3.192	3.065	3.384	3.210	3.910	3.599	3.571	3.768			
MCI Drink	3.806	3.575	3.645	3.649	3.458	3.935	3.218	3.517	3.361	3.477	3.656			
MCI Gel	3.046	3.416	3.191	3.325	3.461	3.258	3.019	3.446	3.256	3.294	3.400			
**SM 18:0_23:0**														
Cheese	0.130	0.139	0.122	0.106	0.119	0.116	0.129	0.115	0.123	0.119	0.132	0.794	0.451	0.689

Hom. Cheese	0.160	0.111	0.149	0.137	0.127	0.129	0.110	0.145	0.135	0.149	0.151			
MCI Drink	0.136	0.149	0.150	0.141	0.123	0.136	0.124	0.127	0.120	0.129	0.138			
MCI Gel	0.107	0.140	0.119	0.136	0.133	0.103	0.109	0.129	0.113	0.125	0.132			
**SM 18:0_24:0**														
Cheese	1.222	1.164	1.191	1.035	1.247	1.116	1.064	1.141	1.269	1.062	1.157	0.794	0.649	0.689
Hom. Cheese	1.351	1.228	1.239	1.134	0.978	1.096	1.060	1.299	1.185	1.217	1.252			
MCI Drink	1.048	1.285	1.339	1.241	1.143	1.378	1.095	1.272	1.033	1.262	1.026			
MCI Gel	1.130	1.349	1.095	1.131	1.254	0.978	1.003	1.030	1.052	1.191	1.148			

*Table S3. Relative level of phosphatidylethanolamine (PE) in plasma samples. Data were analyzed by repeated measures using linear mixed model. Overall participant and within-visit participant differences were included as random factors and the analyses were adjusted for BMI, age and visit. q-values indicate FDR-adjusted p-values for meal-time interactions ($q_{meal*time}$), the effect of meal (q_{meal}) and the effect of time (q_{time}). * indicate significant differences (q < 0.05).*

	0	30	60	90	120	180	240	300	360	420	480	$q_{meal*time}$	q_{meal}	q_{time}
**PE 18:1_16:0**														
Cheese	2.740	2.834	2.435	2.486	2.739	2.769	3.004	2.593	3.293	2.970	3.130	0.794	0.562	0.010*
Hom. Cheese	3.198	2.372	3.117	2.660	2.613	3.003	2.957	3.360	3.051	3.153	3.360			
MCI Drink	2.602	2.658	2.965	2.922	2.872	2.983	2.778	2.868	2.768	2.956	3.425			
MCI Gel	2.414	2.920	2.614	2.654	2.646	2.719	2.638	3.072	2.992	3.145	2.901			
**PE 18:0_16:0**														
Cheese	0.958	1.017	0.861	0.827	1.035	1.063	1.128	0.925	1.245	1.096	1.135	0.690	0.451	0.001*
Hom. Cheese	1.275	0.830	1.010	0.909	0.969	1.093	1.074	1.282	1.174	1.337	1.237			
MCI Drink	0.939	0.948	1.140	1.031	1.011	1.128	0.999	1.075	1.013	1.141	1.219			
MCI Gel	0.883	0.983	0.948	0.910	1.040	0.952	0.942	1.334	1.071	1.112	1.037			
**PE 16:0_18:2**														
Cheese	0.003	0.003	0.003	0.002	0.003	0.003	0.003	0.003	0.003	0.003	0.003	0.281	0.649	0.115
Hom. Cheese	0.003	0.002	0.003	0.002	0.003	0.003	0.003	0.003	0.003	0.004	0.003			
MCI Drink	0.003	0.002	0.003	0.003	0.003	0.004	0.003	0.003	0.002	0.003	0.003			
MCI Gel	0.002	0.003	0.002	0.003	0.003	0.003	0.003	0.003	0.003	0.003	0.002			
**PE 16:0_18:1**														
Cheese	0.080	0.119	0.110	0.119	0.161	0.168	0.179	0.163	0.205	0.141	0.147	0.794	0.564	<0.001*
Hom. Cheese	0.087	0.069	0.112	0.109	0.137	0.162	0.170	0.198	0.144	0.174	0.130			
MCI Drink	0.070	0.075	0.106	0.108	0.135	0.186	0.151	0.173	0.148	0.139	0.149			
MCI Gel	0.082	0.078	0.109	0.125	0.150	0.156	0.173	0.198	0.160	0.152	0.118			
**PE 18:1_18:0**														
Cheese	0.957	1.227	0.991	0.959	1.106	1.124	1.369	1.227	1.581	1.579	1.606	0.826	0.636	<0.001*
Hom. Cheese	1.098	0.874	1.143	1.013	1.057	1.151	1.193	1.574	1.423	1.500	1.587			
MCI Drink	1.007	1.030	1.091	1.094	1.163	1.270	1.255	1.408	1.317	1.577	1.535			
MCI Gel	0.900	1.075	0.943	1.002	1.095	1.141	1.152	1.427	1.367	1.470	1.432			
**PE 16:0_20:4**														
Cheese	53.017	57.307	51.906	48.563	59.892	56.121	58.931	59.369	69.447	60.739	68.412	0.794	0.451	<0.001*
Hom. Cheese	61.113	50.573	63.995	50.759	50.825	57.478	56.898	65.733	64.281	64.382	66.614			
MCI Drink	52.989	50.125	59.369	56.005	57.183	65.967	55.036	64.754	57.382	60.446	64.003			
MCI Gel	48.995	50.552	49.740	52.496	55.114	52.344	52.093	58.255	54.810	62.010	57.733			
**PE 18:1_18:1**														
Cheese	0.004	0.007	0.007	0.007	0.010	0.011	0.010	0.011	0.013	0.010	0.008	0.833	0.644	<0.001*

Hom. Cheese	0.006	0.004	0.008	0.007	0.009	0.010	0.011	0.011	0.010	0.009	0.007			
MCI Drink	0.004	0.005	0.007	0.009	0.010	0.014	0.010	0.010	0.009	0.008	0.009			
MCI Gel	0.006	0.005	0.007	0.008	0.010	0.012	0.012	0.014	0.011	0.011	0.006			
**PE 18:0_18:2**														
Cheese	0.425	0.602	0.619	0.671	0.911	0.936	0.953	0.817	0.992	0.742	0.644	0.813	0.750	<0.001*
Hom. Cheese	0.478	0.398	0.775	0.667	0.868	0.919	0.967	0.964	0.801	0.831	0.618			
MCI Drink	0.375	0.467	0.636	0.805	0.875	1.162	0.881	0.900	0.797	0.730	0.723			
MCI Gel	0.445	0.497	0.686	0.789	0.918	0.926	0.933	1.145	0.849	0.760	0.574			
**PE 18:0_18:1**														
Cheese	0.080	0.128	0.136	0.143	0.182	0.202	0.212	0.184	0.221	0.163	0.133	0.833	0.564	<0.001*
Hom. Cheese	0.089	0.084	0.164	0.137	0.168	0.195	0.203	0.225	0.171	0.185	0.131			
MCI Drink	0.083	0.105	0.138	0.158	0.193	0.244	0.192	0.199	0.185	0.159	0.171			
MCI Gel	0.089	0.106	0.134	0.170	0.197	0.198	0.213	0.254	0.199	0.182	0.138			
**PE 16:0_22:6**														
Cheese	0.221	0.262	0.231	0.244	0.288	0.272	0.262	0.227	0.286	0.236	0.221	0.794	0.174	0.022*
Hom. Cheese	0.234	0.180	0.283	0.244	0.245	0.253	0.272	0.269	0.239	0.272	0.231			
MCI Drink	0.171	0.183	0.246	0.242	0.239	0.265	0.202	0.240	0.212	0.222	0.215			
MCI Gel	0.193	0.226	0.255	0.238	0.246	0.220	0.209	0.246	0.192	0.220	0.201			
**PE 18:0_20:4**														
Cheese	0.329	0.453	0.416	0.438	0.559	0.506	0.565	0.483	0.584	0.495	0.437	0.833	0.562	<0.001*
Hom. Cheese	0.372	0.305	0.475	0.429	0.496	0.544	0.518	0.549	0.478	0.496	0.423			
MCI Drink	0.298	0.322	0.410	0.441	0.506	0.576	0.461	0.478	0.422	0.417	0.449			
MCI Gel	0.318	0.376	0.440	0.442	0.506	0.477	0.504	0.538	0.465	0.463	0.375			
**PE 18:2_22:1**														
Cheese	39.710	42.988	37.436	35.693	43.560	40.425	42.981	42.256	49.570	44.956	48.597	0.833	0.149	0.003*
Hom. Cheese	43.243	35.578	46.275	37.381	37.567	41.949	41.338	47.545	45.366	46.135	47.329			
MCI Drink	39.534	37.862	43.542	42.644	42.207	49.024	41.857	47.900	42.604	45.430	47.766			
MCI Gel	37.099	39.374	36.450	37.992	39.595	39.012	38.943	43.406	40.668	44.707	42.484			
**PE 18:0_22:6**														
Cheese	0.109	0.136	0.126	0.134	0.160	0.142	0.137	0.125	0.162	0.122	0.112	0.794	0.294	<0.001*
Hom. Cheese	0.114	0.097	0.147	0.140	0.130	0.151	0.152	0.147	0.129	0.147	0.111			
MCI Drink	0.090	0.103	0.128	0.130	0.136	0.134	0.110	0.131	0.107	0.121	0.110			
MCI Gel	0.098	0.119	0.135	0.134	0.151	0.141	0.119	0.138	0.115	0.124	0.110			

*Table S4. Relative level of phosphatidylcholine (PC) in plasma samples. Data were analyzed by repeated measures using linear mixed model. Overall participant and within-visit participant differences were included as random factors and the analyses were adjusted for BMI, age and visit. q-values indicate FDR-adjusted p-values for meal-time interactions ($q_{meal*time}$), the effect of meal (q_{meal}) and the effect of time (q_{time}). * indicate significant differences (q < 0.05).*

	0	30	60	90	120	180	240	300	360	420	480	$q_{meal*time}$	q_{meal}	q_{time}
**PC 16:0_16:1**														
Cheese	5.072	6.429	5.195	4.978	5.768	5.895	7.162	6.499	8.269	7.910	8.597	0.833	0.564	<0.001*
Hom. Cheese	5.753	4.494	6.105	5.191	5.331	5.902	6.243	7.941	7.432	7.813	8.242			
MCI Drink	5.122	5.259	5.680	5.494	5.913	6.845	6.473	7.240	6.908	7.992	8.298			
MCI Gel	4.574	5.456	4.953	5.009	5.543	5.854	5.902	7.269	7.080	7.664	7.347			
**PC 16:0_16:0**														
Cheese	9.691	10.666	8.838	8.989	10.020	10.662	12.474	10.998	13.568	12.495	13.222	0.805	0.564	<0.001*
Hom. Cheese	10.758	8.738	10.896	9.163	9.332	10.745	11.036	13.769	12.184	13.256	13.787			
MCI Drink	9.434	9.276	10.107	10.071	10.277	11.412	11.241	11.937	11.556	12.747	13.527			
MCI Gel	8.390	9.716	8.940	9.235	9.537	10.199	10.678	12.783	11.906	12.840	11.714			
**PC 16:0_18:2**														
Cheese	0.003	0.003	0.003	0.002	0.003	0.003	0.003	0.003	0.003	0.003	0.003	0.833	0.164	0.001*
Hom. Cheese	0.003	0.002	0.003	0.002	0.003	0.003	0.003	0.003	0.003	0.004	0.003			
MCI Drink	0.003	0.002	0.003	0.003	0.003	0.004	0.003	0.003	0.002	0.003	0.003			
MCI Gel	0.002	0.003	0.002	0.003	0.003	0.003	0.003	0.003	0.003	0.003	0.002			
**PC 16:0_18:1**														
Cheese	0.080	0.119	0.110	0.119	0.161	0.168	0.179	0.163	0.205	0.141	0.147	0.833	0.562	<0.001*
Hom. Cheese	0.087	0.069	0.112	0.109	0.137	0.162	0.170	0.198	0.144	0.174	0.130			
MCI Drink	0.070	0.075	0.106	0.108	0.135	0.186	0.151	0.173	0.148	0.139	0.149			
MCI Gel	0.082	0.078	0.109	0.125	0.150	0.156	0.173	0.198	0.160	0.152	0.118			
**PC 16:0_20:5**														
Cheese	7.267	7.671	7.812	6.360	7.453	7.653	9.415	8.291	9.414	8.634	9.388	0.833	0.424	<0.001*
Hom. Cheese	8.642	7.502	8.155	7.351	6.508	7.693	7.898	9.940	8.471	9.984	9.000			
MCI Drink	6.176	6.866	7.739	6.835	6.551	8.149	7.295	8.388	7.607	8.182	7.672			
MCI Gel	5.874	6.201	6.680	6.570	7.472	6.772	7.842	8.060	8.084	9.056	7.324			
**PC 16:0_20:4**														
Cheese	53.017	57.307	51.906	48.563	59.892	56.121	58.931	59.369	69.447	60.739	68.412	0.833	0.123	0.001*
Hom. Cheese	61.113	50.573	63.995	50.759	50.825	57.478	56.898	65.733	64.281	64.382	66.614			
MCI Drink	52.989	50.125	59.369	56.005	57.183	65.967	55.036	64.754	57.382	60.446	64.003			
MCI Gel	48.995	50.552	49.740	52.496	55.114	52.344	52.093	58.255	54.810	62.010	57.733			
**PC 16:0_20:3**														
Cheese	36.308	39.683	34.406	34.264	40.785	37.169	39.692	38.950	45.608	40.448	44.691	0.833	0.149	0.015*

Hom. Cheese	40.125	32.712	43.470	35.083	34.249	39.334	37.746	44.598	40.977	42.145	43.873			
MCI Drink	36.841	35.994	40.062	39.637	39.498	44.632	39.277	45.303	39.322	42.059	43.593			
MCI Gel	34.328	37.643	33.424	36.397	37.211	36.100	36.261	41.057	37.225	40.547	38.605			
**PC 18:0_18:2**														
Cheese	62.528	66.782	59.592	58.044	68.535	64.647	68.489	67.666	79.597	69.881	75.162			
Hom. Cheese	72.295	58.446	74.605	62.109	62.004	69.370	68.449	80.272	75.143	75.214	79.318	0.833	0.149	0.001*
MCI Drink	62.839	63.981	71.910	70.142	70.584	80.326	69.793	80.915	71.258	75.330	77.505			
MCI Gel	59.248	63.692	58.446	63.375	66.344	64.022	65.910	72.556	66.974	75.973	70.297			
**PC 18:0_18:1**														
Cheese	10.063	11.722	9.892	9.504	11.278	10.647	11.742	11.232	13.710	12.394	13.594			
Hom. Cheese	11.796	9.269	11.838	10.103	9.614	10.601	11.082	13.276	12.670	12.951	13.184	0.794	0.564	<0.001*
MCI Drink	10.075	9.954	11.349	11.320	10.891	13.128	11.358	12.884	11.775	12.952	12.651			
MCI Gel	9.613	10.346	9.989	10.332	10.937	10.354	10.841	12.370	11.398	13.065	12.134			
**PC 18:1_20:4**														
Cheese	22.339	24.184	21.813	20.986	24.327	23.586	25.371	24.012	29.151	25.502	27.689			
Hom. Cheese	25.147	21.602	26.453	22.323	20.985	24.125	24.540	29.716	26.066	28.699	27.587	0.833	0.553	0.001*
MCI Drink	21.489	21.505	24.650	23.075	23.667	26.302	23.154	26.668	23.934	25.408	26.315			
MCI Gel	20.371	21.866	21.521	22.479	24.124	22.226	22.567	24.615	24.081	26.155	24.755			

Table S5. List of investigated metabolites. CER, ceramide; LPC, lysophosphatidylcholine; LPE, lysophosphatidylethanolamine; PC, phosphatidylcholine; PE, phosphatidylethanolamine; SM, sphingomyelin.

	Class	Species
**1**	CER	CER 18:0_18:1
**2**		CER 18:0_18:2
**3**		CER 18:0_20:2
**4**		CER 18:0_22:1
**5**		CER 18:0_24:0
**6**		CER 18:0_26:2
**7**		CER 18:0_26:3
**8**	LPC	LPC 16:3
**9**		LPC 18:0
**10**		LPC 18:1
**11**		LPC 18:4
**12**		LPC 20:0
**13**		LPC 22:0
**14**		LPC 24:0
**15**	LPE	LPE 16:0
**16**		LPE 18:0
**17**		LPE 18:1
**18**		LPE 18:2
**19**		LPE 20:1
**20**		LPE 20:2
**21**		LPE 20:3
**22**		LPE 22:6
**23**	PC	PC 16:0_16:0
**24**		PC 16:0_16:1
**25**		PC 16:0_18:1
**26**		PC 16:0_18:2
**27**		PC 16:0_20:3
**28**		PC 16:0_20:4
**29**		PC 16:0_20:5
**30**		PC 18:0_18:1
**31**		PC 18:0_18:2
**32**		PC 18:1_20:4
**33**	PE	PE 16:0_18:1
**34**		PE 16:0_18:2
**35**		PE 16:0_20:4
**36**		PE 16:0_22:6
**37**		PE 18:0_16:0
**38**		PE 18:0_18:1
**39**		PE 18:0_18:2
**40**		PE 18:0_20:4
**41**		PE 18:0_22:6
**42**		PE 18:1_16:0
**43**		PE 18:1_18:0

**44**		PE 18:1_18:1
**45**		PE 18:2_22:1
**46**	SM	SM 18:0_14:0
**47**		SM 18:0_20:0
**48**		SM 18:0_22:0
**49**		SM 18:0_22:1
**50**		SM 18:0_23:0
**51**		SM 18:0_24:0
**52**		SM 18:1_22:0
**53**		SM 18:1_23:0

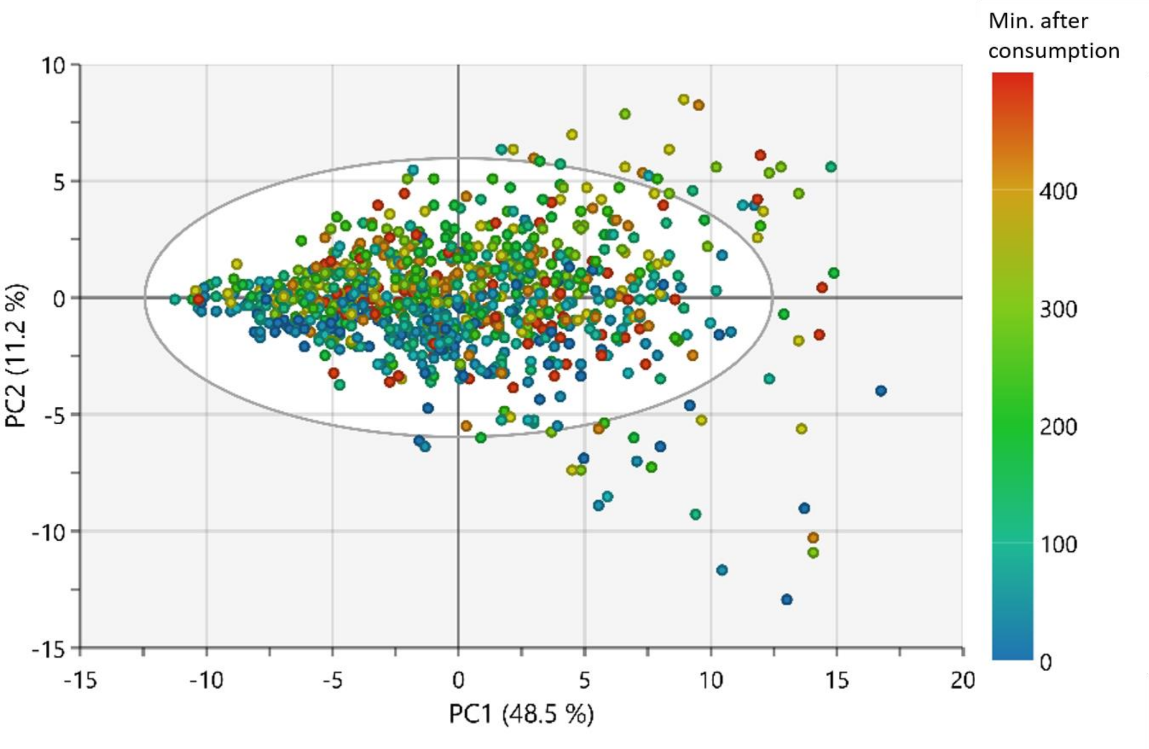

Figure S1. PCA scores plot of phospholipid content in plasma samples obtained from LC-MS. Colors indicate time after consumption of test meals.

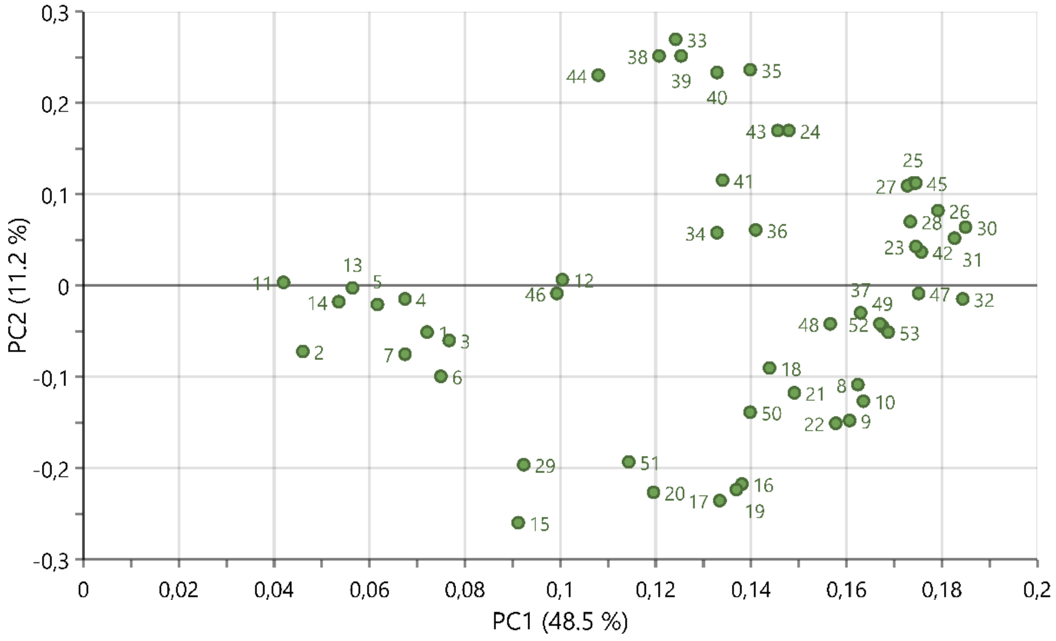

Figure S2. Corresponding PCA loadings plots containing 53 phospholipids. The phospholipids belonging to each of the numbers can be seen in Table S5.

1)

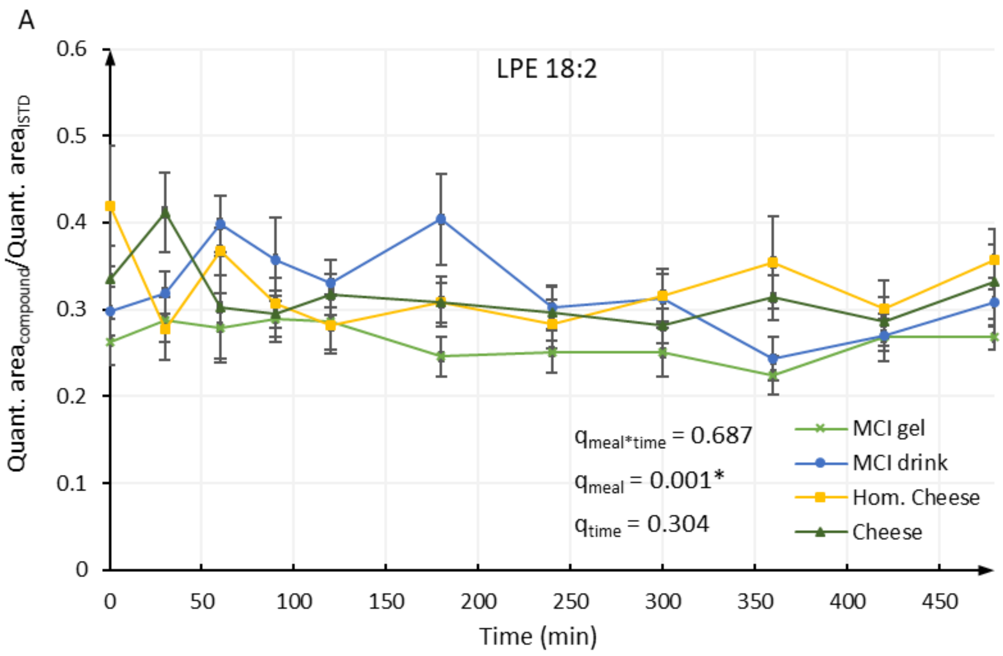

B

$P_{MCI\ drink\ vs.\ MCI\ gel} < 0.001^{\#}$

$P_{Hom.\ Cheese\ vs.\ MCI\ gel} < 0.001^{\#}$

$P_{Cheese\ vs.\ MCI\ gel} < 0.001^{\#}$

$P_{Hom.\ Cheese\ vs.\ MCI\ drink} = 0.98$

$P_{Cheese\ vs.\ MCI\ drink} = 0.99$

$P_{Cheese\ vs.\ Hom.\ Cheese} = 1.00$

2)

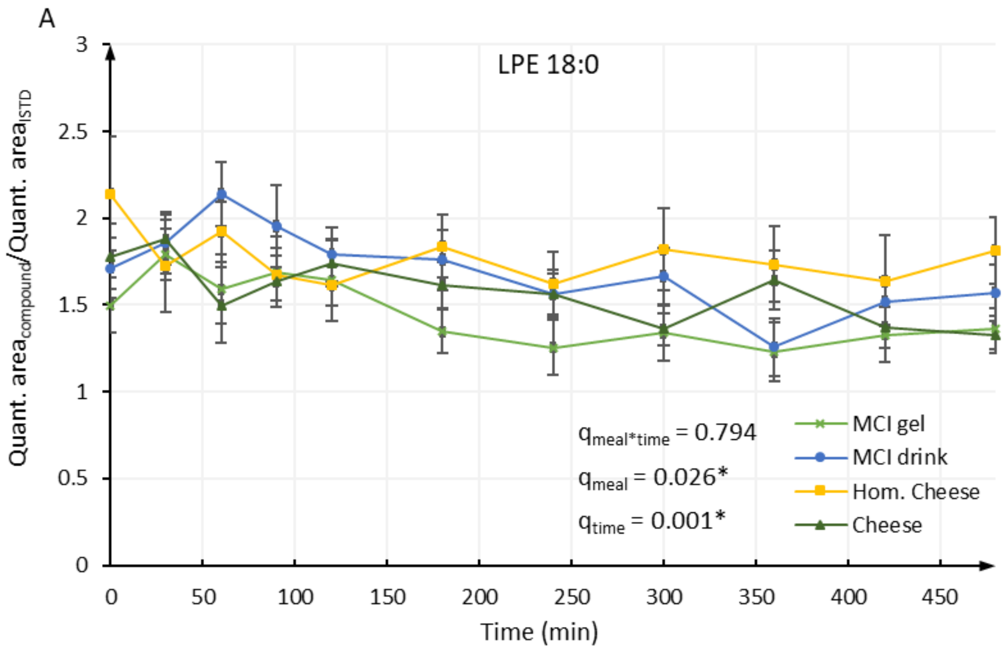

B

$P_{MCI\ drink\ vs.\ MCI\ gel} = 0.012^{\#}$

$P_{Hom.\ Cheese\ vs.\ MCI\ gel} = 0.004^{\#}$

$P_{Cheese\ vs.\ MCI\ gel} = 0.62$

$P_{Hom.\ Cheese\ vs.\ MCI\ drink} = 0.98$

$P_{Cheese\ vs.\ MCI\ drink} = 0.26$

$P_{Cheese\ vs.\ Hom.\ Cheese} = 0.12$

3)

A

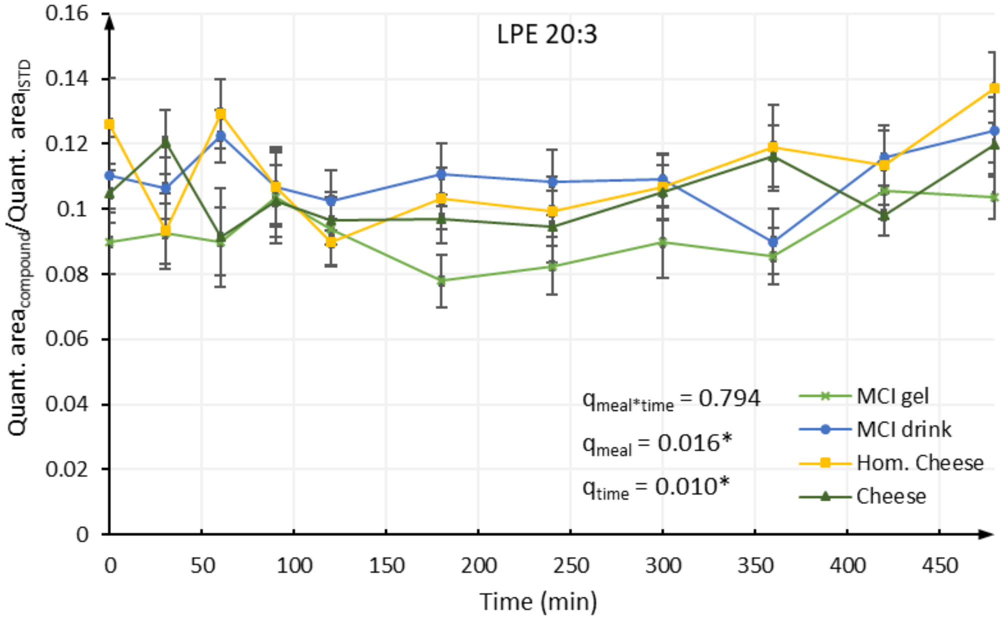

B

$P_{MCI\ drink\ vs.\ MCI\ gel}$ = 0.003#

$P_{Hom.\ Cheese\ vs.\ MCI\ gel}$ = 0.001#

$P_{Cheese\ vs.\ MCI\ gel}$ = 0.054

$P_{Hom.\ Cheese\ vs.\ MCI\ drink}$ = 1.00

$P_{Cheese\ vs.\ MCI\ drink}$ = 0.77

$P_{Cheese\ vs.\ Hom.\ Cheese}$ = 0.63

4)

A

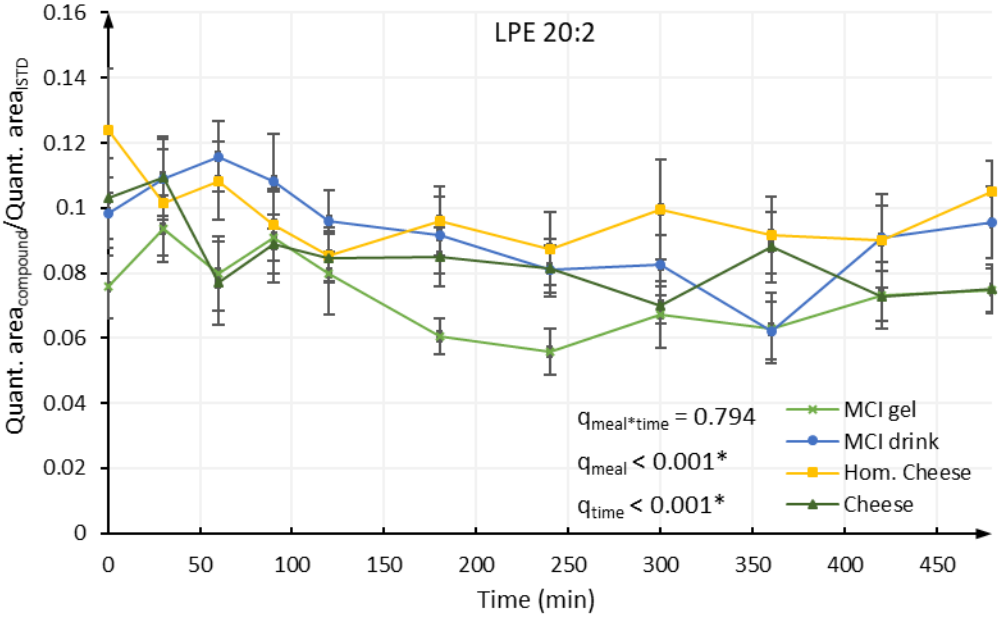

B

$P_{MCI\ drink\ vs.\ MCI\ gel}$ < 0.001#

$P_{Hom.\ Cheese\ vs.\ MCI\ gel}$ < 0.001#

$P_{Cheese\ vs.\ MCI\ gel}$ = 0.13

$P_{Hom.\ Cheese\ vs.\ MCI\ drink}$ = 0.91

$P_{Cheese\ vs.\ MCI\ drink}$ = 0.13

$P_{Cheese\ vs.\ Hom.\ Cheese}$ = 0.021#

5)

A

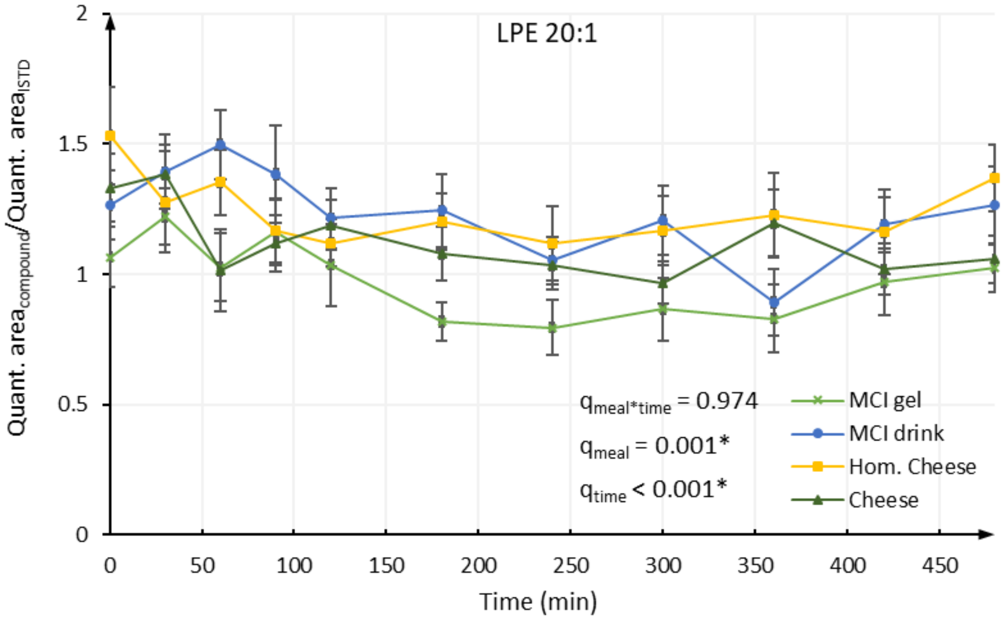

B

$P_{MCI\ drink\ vs.\ MCI\ gel} < 0.001^{\#}$

$P_{Hom.\ Cheese\ vs.\ MCI\ gel} < 0.001^{\#}$

$P_{Cheese\ vs.\ MCI\ gel} = 0.070$

$P_{Hom.\ Cheese\ vs.\ MCI\ drink} = 1.00$

$P_{Cheese\ vs.\ MCI\ drink} = 0.13$

$P_{Cheese\ vs.\ Hom.\ Cheese} = 0.14$

6)

A

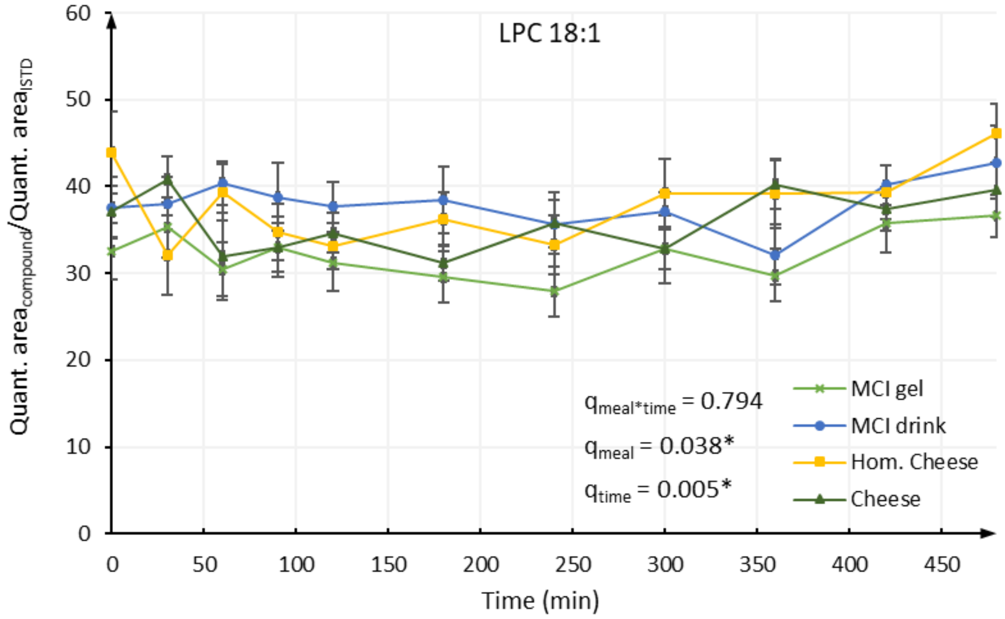

B

$P_{MCI\ drink\ vs.\ MCI\ gel} = 0.005^{\#}$

$P_{Hom.\ Cheese\ vs.\ MCI\ gel} = 0.009^{\#}$

$P_{Cheese\ vs.\ MCI\ gel} = 0.12$

$P_{Hom.\ Cheese\ vs.\ MCI\ drink} = 1.00$

$P_{Cheese\ vs.\ MCI\ drink} = 0.70$

$P_{Cheese\ vs.\ Hom.\ Cheese} = 0.77$

7)

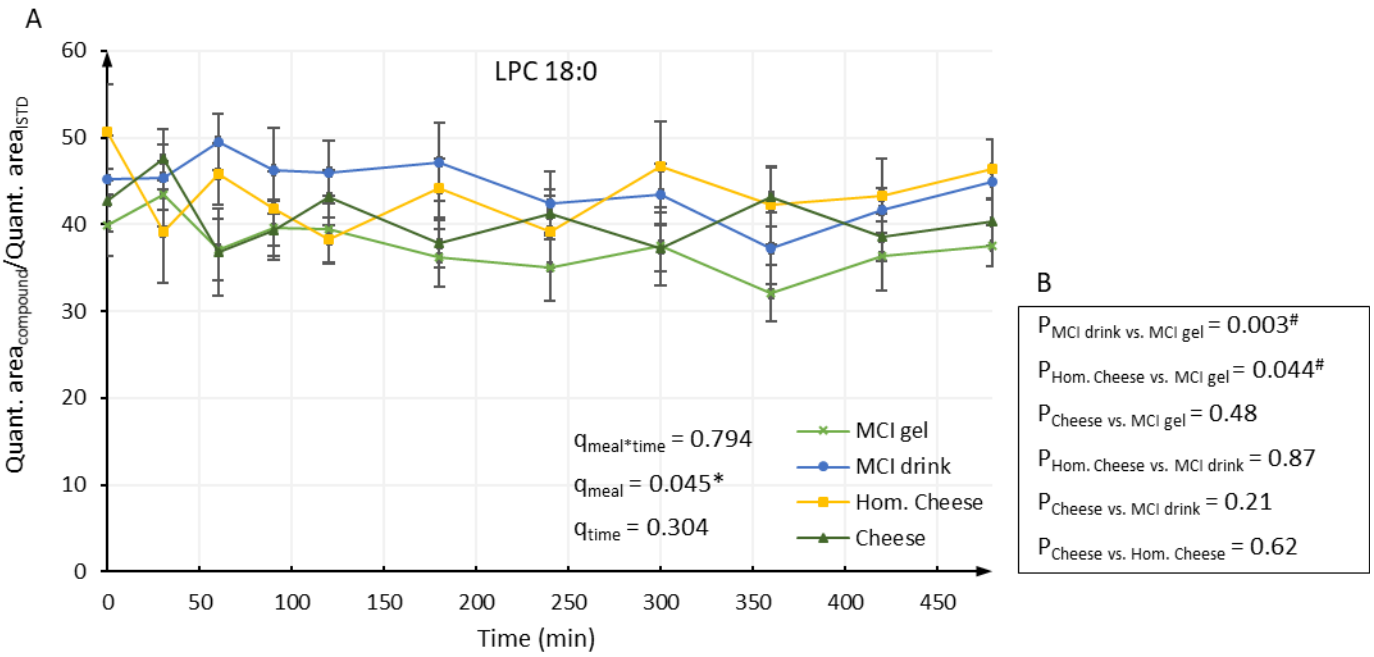

*Figure S3. Relative level of phospholipids in plasma samples for 1) LPE 18:2, 2) LPE 18:0, 3) LPE 20:3, 4) LPE 20:2, 5) LPE 20:1, 6) LPC 18:1, 7) LPC 18:0. A) Data were analyzed by repeated measures using linear mixed model. Overall participant and within-visit participant differences were included as random factors and the analyses were adjusted for BMI, age and visit. q-values indicate FDR-adjusted p-values for meal-time interactions ($q_{meal*time}$), the effect of meal (q_{meal}) and the effect of time (q_{time}). * indicate significant differences ($q < 0.05$). B) p-values obtained from Tukey's all-pairwise comparison conducted when a significant meal effect was observed in repeated measures analysis. #indicate significant differences ($p < 0.05$).*
